# Impact of the recent revision of arvc task force criteria for cmr on criteria prevalence and diagnostic accuracy

**DOI:** 10.1186/1532-429X-13-S1-P322

**Published:** 2011-02-02

**Authors:** Emmanuelle Vermes, Oliver Strohm, Akli Otmani, Helene Childs, Hank Duff, Matthias G Friedrich

**Affiliations:** 1 Calgary, calgary, AB, Canada

## Background

Recently, the ESC Task Force Criteria (TFC) for arrythmogenic right ventricular cardiomyopathy (ARVC) have been revised, aiming for a better diagnostic sensitivity. We attempted to assess the impact of the revision on the prevalence and diagnostic accuracy of CMR criteria.

## Methods

We retrospectively evaluated the CMR scans of 294 patients referred for ARVC between 2005 and 2010 and determined the presence or absence of major and minor CMR criteria using the original and the revised TFC. Previously, major and minor abnormalities were identified by the presence of RV dilatation (global or segmental), RV micro-aneurysm, or regional hypokinesis. The revised criteria require the combination of severe regional wall motion abnormalities (akinesis or dyskinesis or dyssynchrony) with global RV dilatation or dysfunction (quantitative assessment). For defining RV dilatation, we used the same quantitative cut-off values for both, original and revised criteria.

## Results

Applying the original criteria, 69 patients (23,4%) had major original criteria vs. 19 patients (6,5%) with the revised (p<0.001). Forty-three patients (62,3%) with major original criteria did not meet any of the revised criteria.

Using the original criteria, 172 patients (58,5%) had at least 1 minor criterion vs. 12 patients (4%) with the revised TFC (p<0.001); 168 patients (97,5%) with minor original criteria did not have any of the revised criteria.

The difference was mainly due to findings of regional wall motion abnormalities or microaneurysms in the absence of RV dilatation, qualifying as a criterion according to the original, but not the revised criteria.

In the subgroup of 134 patients with complete diagnostic work-up of ARVC, ten patients met the diagnosis of proven ARVC without counting imaging criteria. Only 4/10 had major criteria according to the revised CMR criteria; none had minor criteria. However, 112/124 patients without ARVC were correctly classified as negative by major and minor criteria (specificity 94% and 96%, respectively).

## Conclusion

In our experience, the revision of the ARVC Task Force imaging criteria significantly reduced the overall prevalence of major and minor criteria. The required combination of regional wall motion criteria with global RV dilatation/dysfunction may misdiagnose patients with ARVC, possibly related to the fact that ARVC tissue abnormalities may occur predominantly locally. The revision, although maintaining a high specificity, may not have improved the sensitivity for identifying patients with ARVC. Larger studies including follow-up are required.

